# Effects of Neurolytic Celiac Plexus Block on Liver Regeneration in Rats with Partial Hepatectomy

**DOI:** 10.1371/journal.pone.0073101

**Published:** 2013-09-05

**Authors:** Jun Li, Hong-Tao Yan, Jian-Xiang Che, Shu-Rong Bai, Qing-Ming Qiu, Ling Ren, Fan Pan, Xiao-Qin Sun, Fu-Zhou Tian, Dong-Xuan Li, Li-Jun Tang

**Affiliations:** 1 Department of Anesthesia, General Hospital of Chengdu Military Command Area, Chengdu, Sichuan Province, P. R. China; 2 General Surgery Center of PLA, General Hospital of Chengdu Military Command Area, Chengdu, Sichuan Province, P. R. China; 3 Department of Anesthesia, the 44^th^ Hospital of PLA, Guiyang, Guizhou Province, P. R. China; University of San Francisco, United States of America

## Abstract

Liver regeneration is the basic physiological process after partial hepatectomy (PH), and is important for the functional rehabilitation of the liver after acute hepatic injury. This study was designed to explore the effects of neurolytic celiac plexus block (NCPB) on liver regeneration after PH. We established a model of PH in rats, assessing hepatic blood flow, liver function, and serum CRP, TNF-α, IL-1β and IL-6 concentrations of the residuary liver after PH. Additionally, histopathological studies, immunohistochemistry, and western blotting were also performed. Our results indicated that NCPB treatment after PH improved liver regeneration and survival rates, increased hepatic blood flow, reduced hepatocyte damage, decreased the secretion and release of inflammatory cytokines, increased the expression of B cell lymphoma/leukemia-2 (Bcl-2), and decreased the expression of Bcl-2 associated X protein (Bax). Additionally, Western blotting revealed that the expression of NF-κB p65 and c-Jun were decreased in liver after NCPB. In conclusion, the results of our present study indicate that NCPB treatment has a favorable effect on liver regeneration after PH. We suggest that NCPB can be utilized as an effective therapeutic method to help the functional rehabilitation of the liver after acute hepatic injury or liver cancer surgery.

## Introduction

The liver is the largest intra-abdominal organ for maintaining vital movement, and there is currently no way to compensate for the absence of liver function long term. In addition, the liver is an indispensable organ for survival with a wide range of functions, including detoxification, glycogen storage, decomposition of sugar, protein & fat synthesis and metabolism, bile secretion, plasma protein synthesis, hormone production, and blood volume regulation [1]. After severe liver injury or surgical resection, liver cells must regenerate to compensate for the lost tissue. Additionally, due to the injury, pain, blood loss, shock, acidosis and other stimuli after severe injury or hepatic cancer surgery, intense stress and inflammatory responses can be induced, causing obvious stress disorder [2]. It has been reported that post-traumatic stress disorder is an important and key initiating factor of secondary injury to the body, including systemic inflammatory response syndrome (SIRS) and multiple organ dysfunction syndrome [3]. After severe trauma, SIRS can be induced by the massive release of inflammatory cytokines and activation of inflammatory mediators. Furthermore, T lymphocyte-mediated immune function is significantly inhibited, so the body’s capacity for defending against infection is lower, leading to an increased susceptibility to infection and multiple organ failure [4]. As such, mechanisms to promote liver tissue regeneration and inhibit the development of SIRS are important for decreasing mortality after sever liver injury or liver cancer surgery.

The celiac plexus, also called the solar plexus, is a complex network of nerves. Currently, neurolytic celiac plexus block (NCPB) is widely used to treat visceral pain in patients with intra-abdominal malignancies, and is especially effective for pain associated with pancreatic cancer, retroperitoneal tumors, and metastatic carcinoma [5]–[7]. Additionally, it is reported that the autonomic nervous system plays an important role in post-traumatic stress and the inflammatory response of the body after severe trauma, such that severe trauma may induce over-excitation of the sympathetic nervous system and generate secretion of inflammatory cytokines, leading to the development of SIRS [8]. The parasympathetic nervous system can also strongly regulate the inflammatory response via acetylcholine; for example, the systemic shock-like reaction induced by endotoxin can be inhibited by stimulating the vagus nerve, and the release of IL-1 and TNF-α can be suppressed by activation of the acetylcholine receptor in macrophages [9]. The celiac plexus is the body’s largest independent plexus; however, the effect of NCPB on severe liver injury is still unclear. Therefore, this study was designed to explore the effect of NCPB on liver regeneration after partial hepatectomy (PH) in a rat model, which has significant reference value for the clinical treatment of severe liver trauma and infection.

## Results

### Effect of NCPB on Survival of Rats after PH

**Figure 1:**
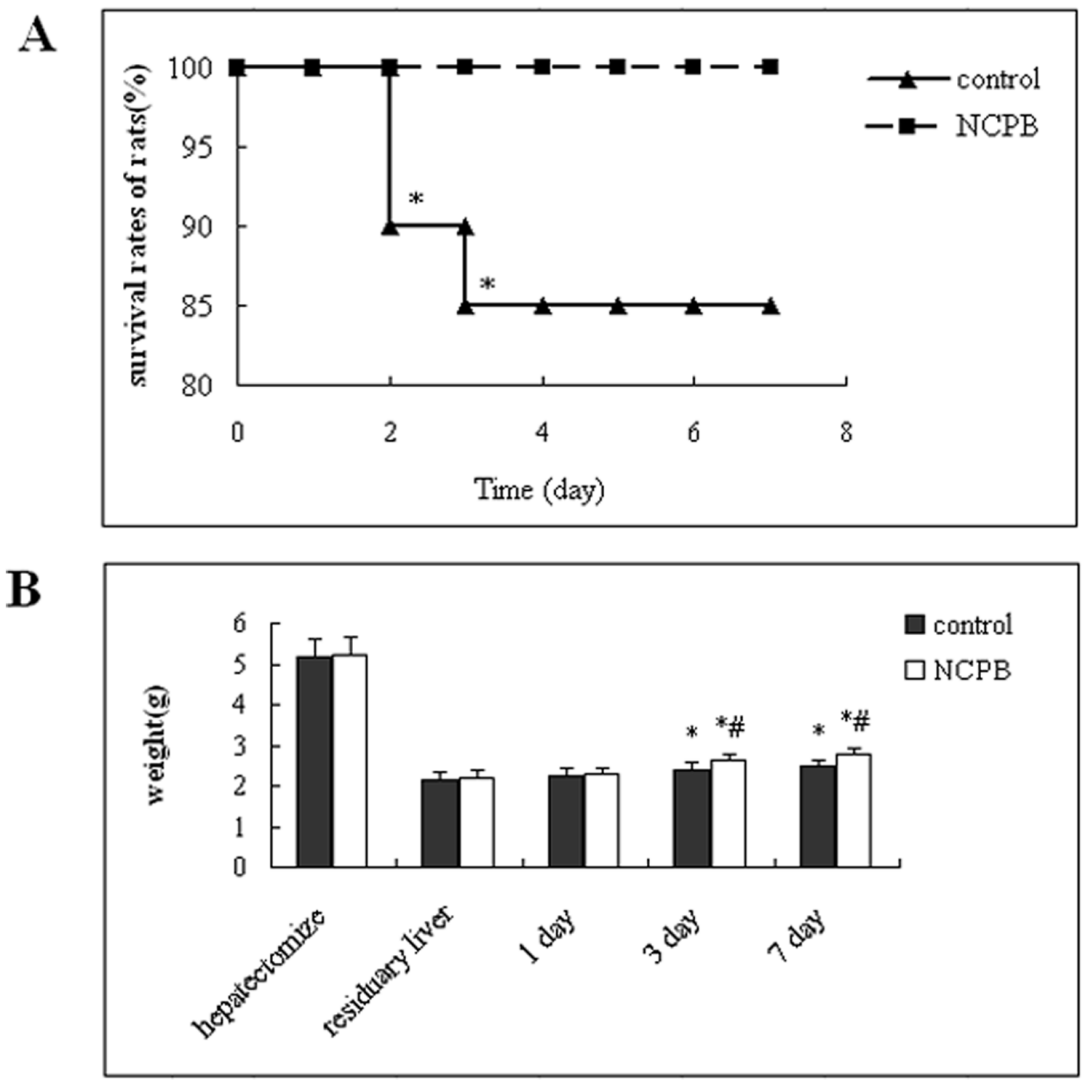
Effect of NCPB on survival and liver regeneration of rats after PH. (**A**) Rats were divided into 2 groups (n = 20). The rats were observed for 7 days. Asterisks indicated significant difference from control group, *p<0.05. (**B**) The weight of residuary liver group was estimated by the formula: hepatectomize/0.705×0.295 [In our present study, the weight ratio of liver resection rate (%) was 70.5±4.4], *P<0.01, compared with the residuary liver group; ^#^P<0.01, compared with the control group.

As can be seen from **Fig. 1** **A**, rat survival in the control group, was 90% at 2 days after surgery and diminished progressively until the end of the observation period, at which time 85% of controls were alive. In the group of rats treated with NCPB, we observed no deaths. This result revealed that NCPB treatment can significantly improve the survival rate after PH (p<0.05).

### Effect of NCPB on Liver Regeneration of Rats after PH

In our present study, the weight ratio of liver/body and liver resection rate were determined, and the values (%) were 3.61±0.12 and 70.5±4.4 (n = 10). Based on the results above, liver regeneration of rat after PH was determined, and the results were shown in the **Fig. 1** **B**. The weight of liver was obviously increased at 3 and 7 days after PH (p<0.05); in addition, the liver weight of NCPB group was significantly higher than that of control group (p<0.05). This result indicated that NCPB treatment can significantly improve the liver regeneration of rats after PH.

### Hepatic Blood Flow of Rats after PH

**Figure 2:**
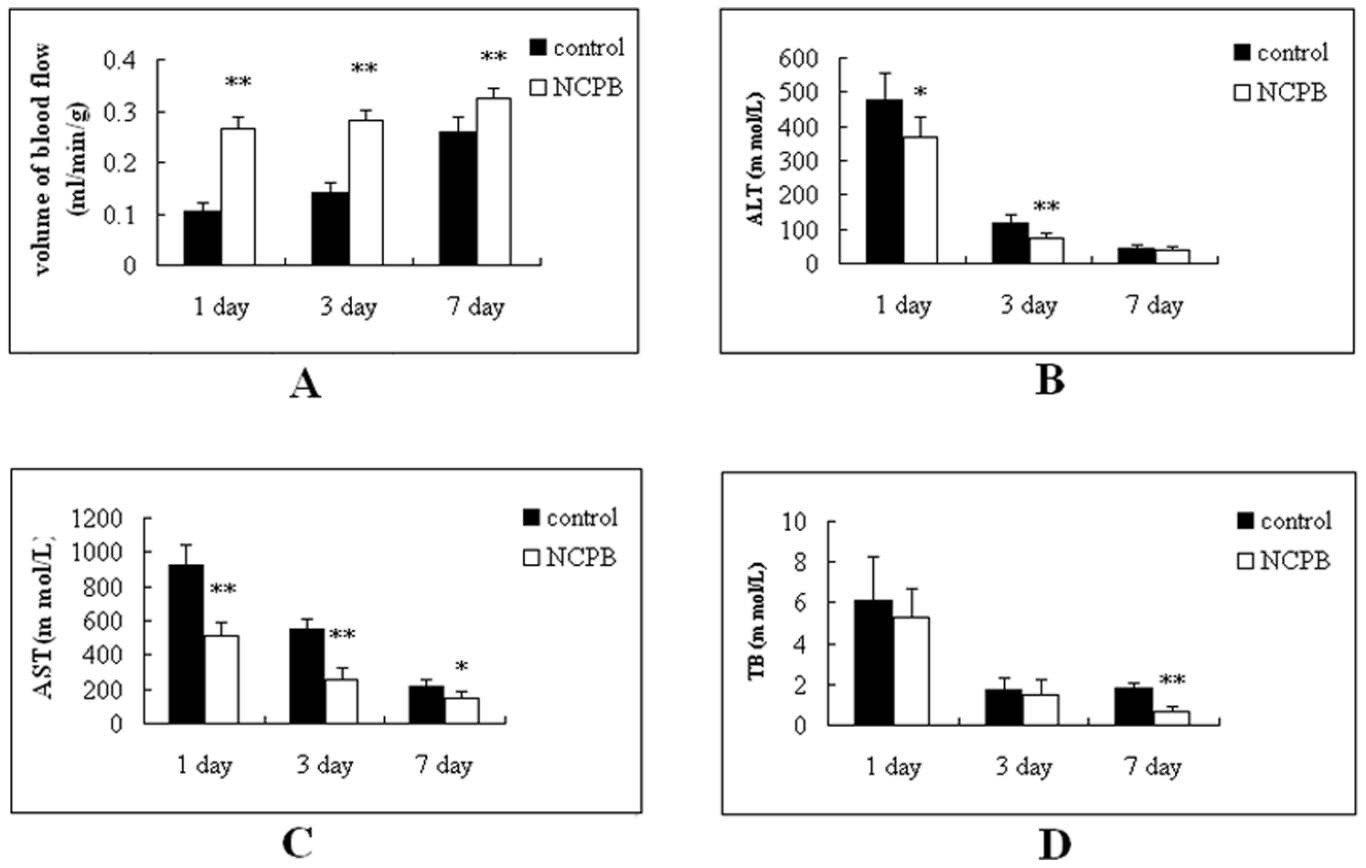
Hepatic blood flow and liver function after PH. Rats were divided into 2 groups (n = 10). The rats were observed at 1, 3, and 7 days after PH. Asterisks indicate significant difference from control group, *p<0.05; **p<0.01.

The radioactive microsphere method was used to determine the hepatic blood flow of rats after PH. As can be seen from **Fig. 2A**, the hepatic blood flow volume of rats in the control group was lowest at 1 day after PH surgery, and increased progressively until the end of the observation period; however, the hepatic blood flow of rats treated with NCPB was higher significantly than that of rats in the control group at each time point (p<0.01). This result revealed that NCPB treatment can significantly improve hepatic blood flow after PH.

### Liver Function of Rats after PH

Liver function was assessed in rats after PH (**Fig. 2B–D**). The serum concentration of alanine transarninase (ALT) in the NCPB group was significantly lower at 1 (p<0.05) and 3 (p<0.01) days after PH, compared to the control group (**Fig. 2B** **)**. Additionally, the serum glutamic-oxalacetic transaminase (AST) concentration in the NCPB group was significantly lower than that of rats in the control group at 1 (p<0.01), 3 (p<0.01) and 7 (p<0.05) days after PH (**Fig. 2C** **)**. The serum concentration of total bilirubin (TB) was also determined (**Fig. 2D** **)**; however, there was no difference between the control and NCPB group at 1 and 3 days after PH, although the TB concentration was significantly lower in NCPB treated rats after 7 days, compared to control (p<0.01).

### Serum Concentration of CRP, TNF-α, IL-1β and IL-6 after PH

**Figure 3:**
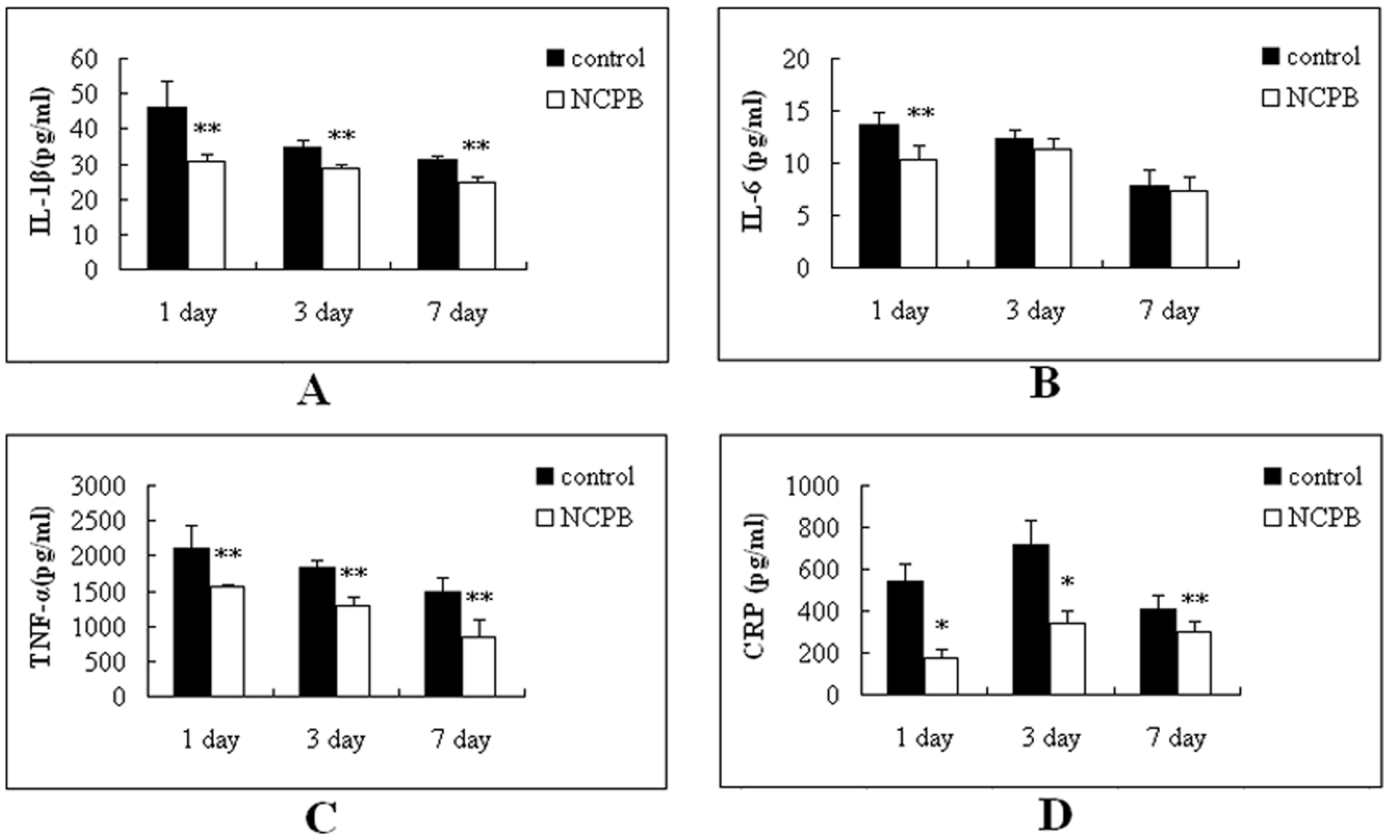
Circulating levels of CRP, TNFα, IL1β and IL6 in rats after PH. Rats were divided into 2 group (n = 10). The rats were observed at 1, 3, and 7 days after PH. Asterisks indicate significant differences from control group. *p<0.05; **p<0.01.

The concentration of inflammatory cytokines was measured in the serum of rats after PH (**Fig. 3**). The concentrations of all the inflammatory cytokines were at their maximum at 1 day after PH, and diminished progressively until the end of the observation period. The serum concentration of IL-1β and TNF-α level in the NCPB group was significantly lower than the control group at each time point (p<0.01; **Fig. 3A and 3C**); Additionally, the IL-6 concentration in the NCPB group was significantly lower than that the control group at 1 day after PH (p<0.01; **Fig. 3B**). The serum concentration of CRP was also determined after PH (**Fig. 3D**). The results indicated that CRP concentration progressively increased in both groups until 3 days after PH, and then decreased from then on until the 7^th^ day after PH. Despite this similar trend, the serum concentration of CRP in the NCPB group was significantly lower significantly than that of the control group at each time point (1 day, p<0.05; 3 days, p<0.05; 7 days, p<0.01).

### Histopathology

**Figure 4:**
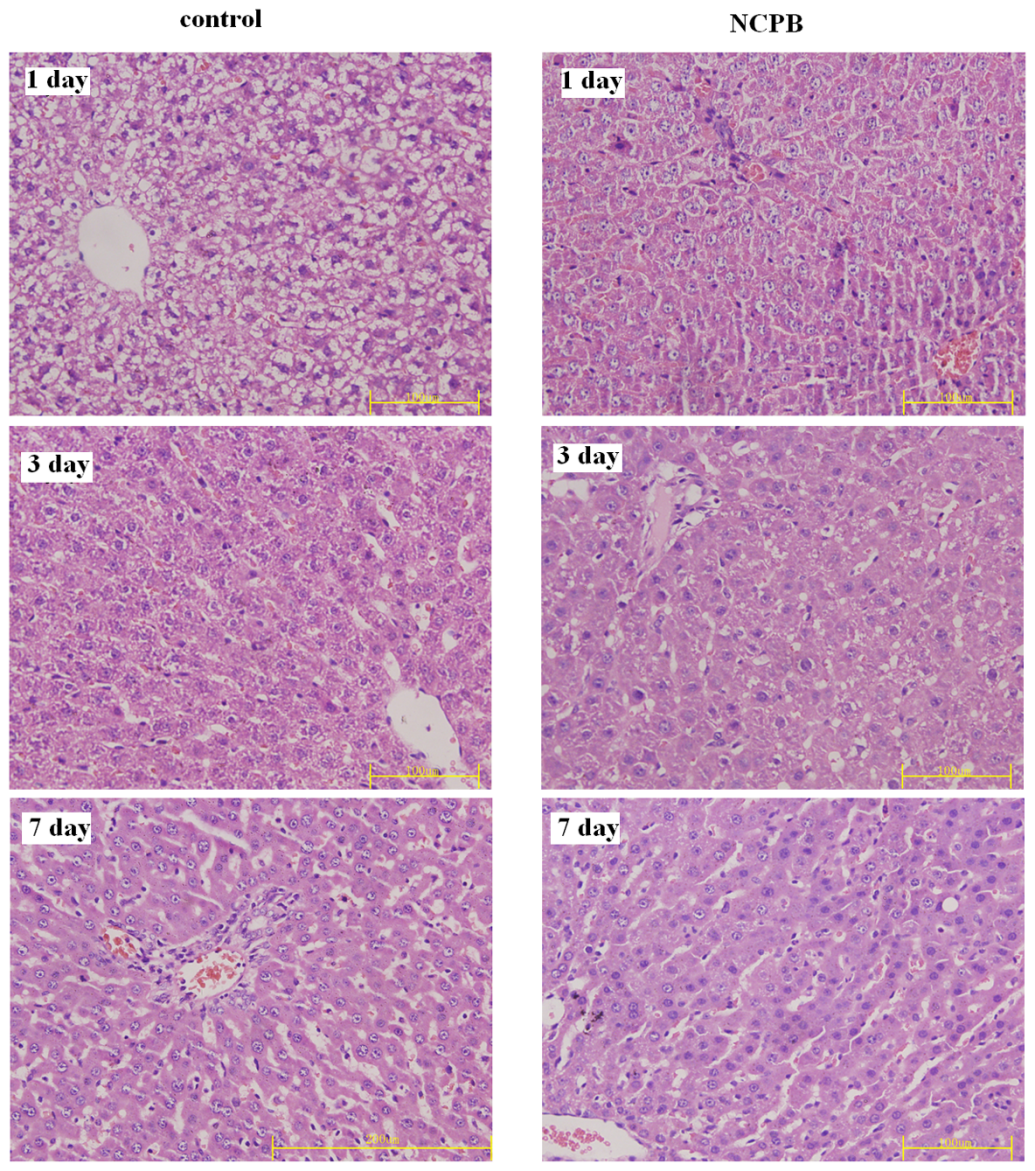
Histopathology of the liver after PH (×40).

Histophathological examination of liver tissue from control and NCPB treated rats was performed after PH (**Fig. 4**). Our microscopic examination revealed that the color of hepatic tissue in the control group became paler 1 day after PH, with hepatocellular cloudy swelling, fatty changes, small pieces and focal necrosis being observed by *H* & *E* staining. In the NCPB treated group, the color of the hepatic tissue was rosy, and there was no obvious fatty change or necrosis observed. Three days after surgery, both hepatic tissues of the control and NCPB groups were rosy. The hepatocellular swelling seen on control rats was lessened and there was no obvious fatty change; however, small necrotic foci were abundant. In the NCPB treated group, there was no obvious fatty change or necrosis, and lots of nucleolus divisions were apparent in the hepatic tissues. Seven days after PH, hepatic tissue in both groups was rosy and arranged in neat rows; however, more mitotic hepatocytes and nuclear divisions were observed in the NCPB group than that of the control group.

### Hepatic Expression of VEGF, Bax and Bcl-2

**Figure 5:**
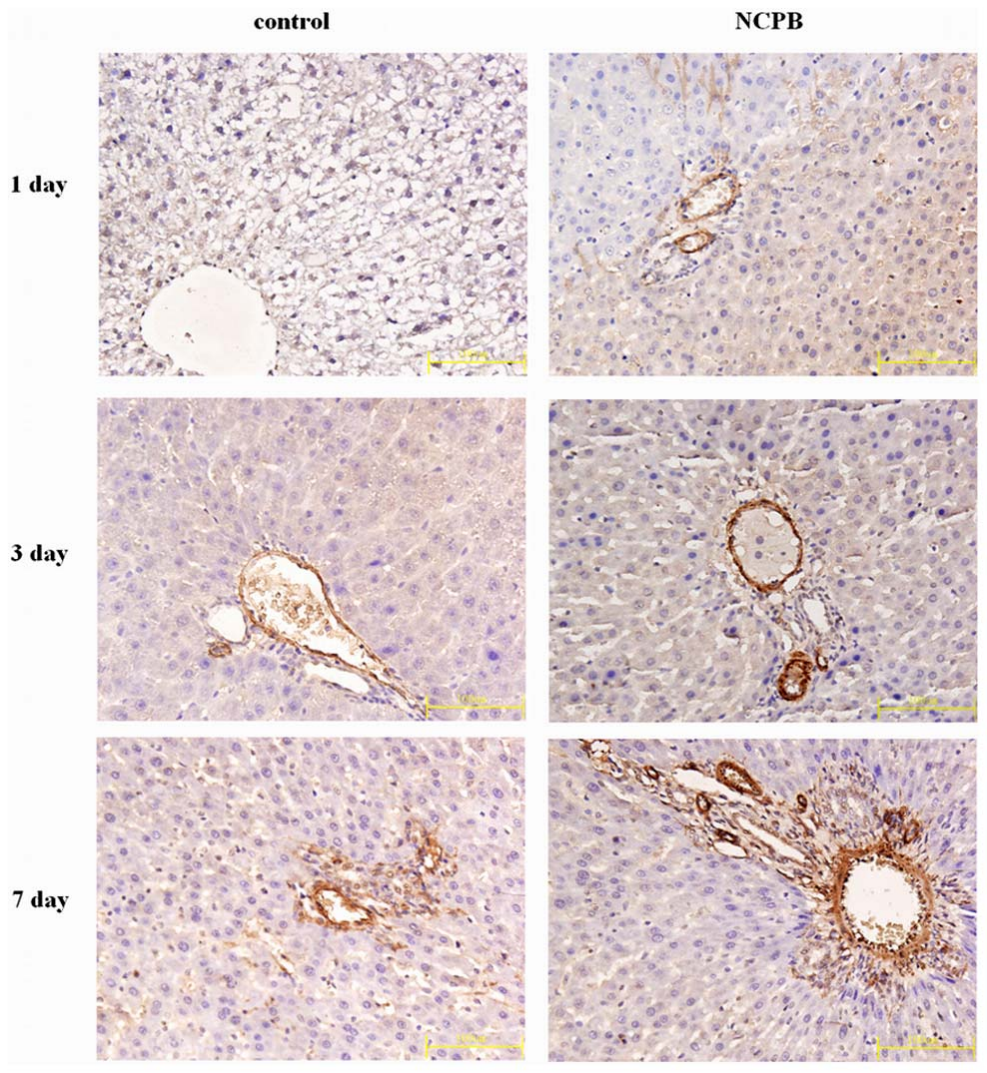
Expressions of VEGF in the liver tissues. (×40).

**Figure 6:**
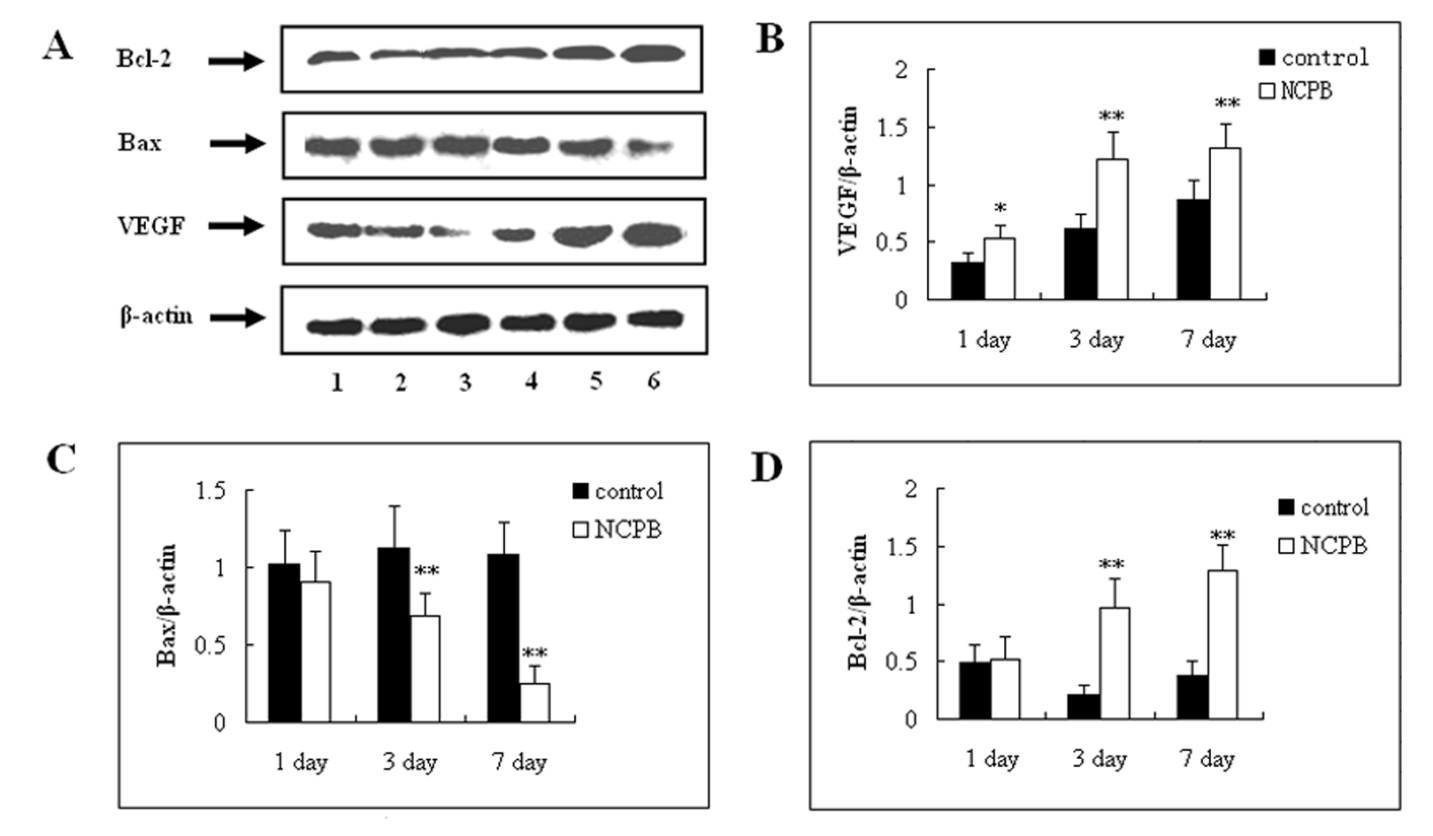
Protein expression level of VEGF, Bax and Bcl-2 proteins in the liver after PH. (A) Lanes 1–3 represent the protein expression level in the control group at 7, 3 and 1 days after PH, respectively. Lanes 4–6 represent the protein expression level in NCPB group at 1, 3 and 7 days after PH, respectively. The expression of VEGF, Bax, and Bcl-2 were detected by Western blot analysis and normalized to response to β-actin. (B–D) represent the statistical charts of VEGF, Bax, and Bcl-2 expressions in the liver after PH, respectively, and asterisks indicate significant differences from control group. *p<0.05; **p<0.01.

**Figure 7:**
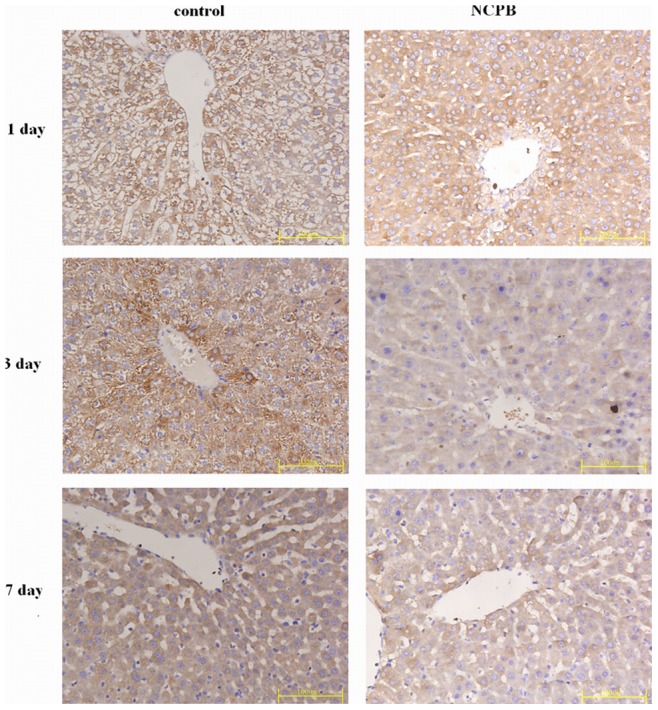
Expressions of Bax in the liver tissues. (×40).

**Figure 8:**
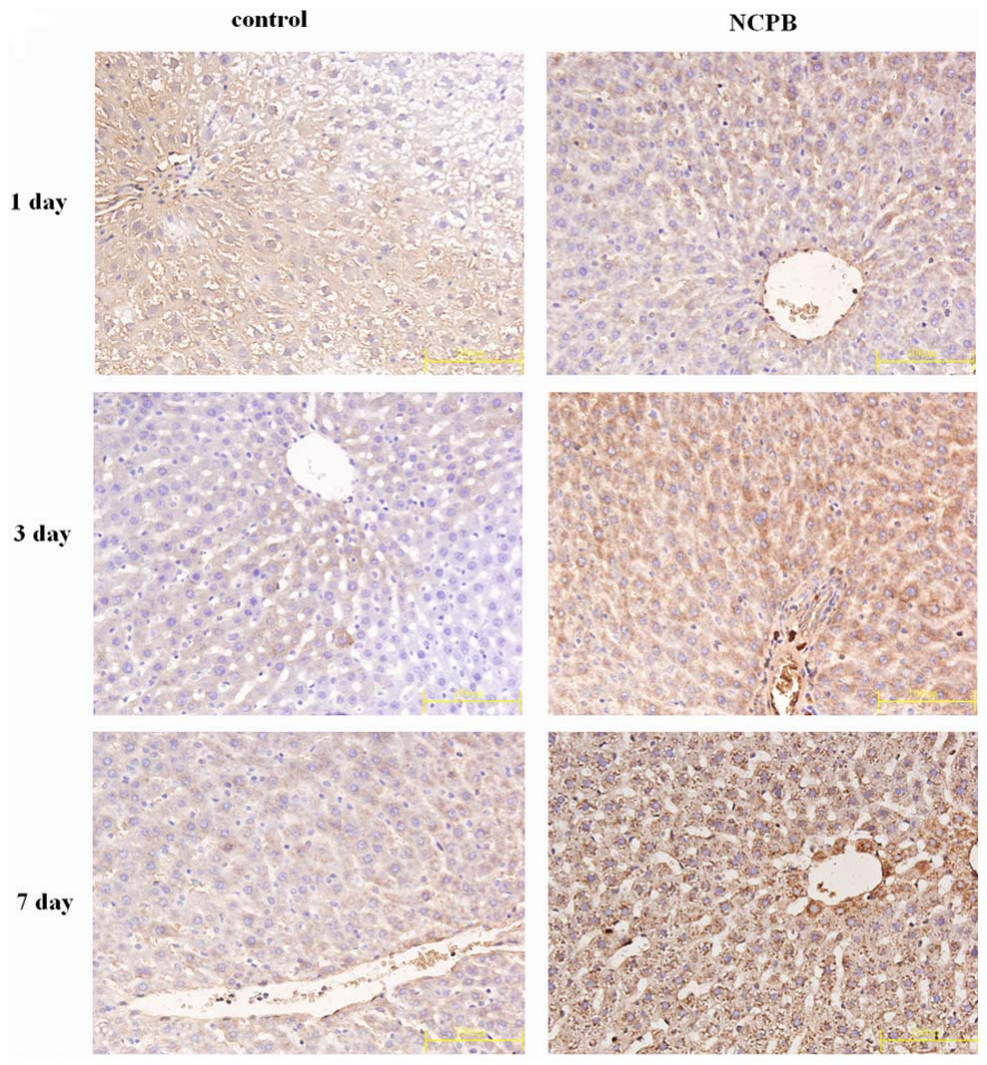
Expressions of Bcl2 in the liver tissues. (×40).

Immunohistochemical staining and western blotting of VEGF, Bax and Bcl-2 was performed in liver sections from control and NCPB treated rats at multiple time points after PH. VEGF immunoreactivity was primarily located in the vascular endothelial cytoplasm. The expression of VEGF was low 1 day after PH, and increased progressively throughout the observation period. The expression of VEGF in NCPB-treated rats was higher than that of control rats at each time point (p<0.05) (**Fig. 5** **&** **Fig. 6** **A, B**). The expression level of Bcl-2 associated X protein (Bax) was also determined. The expression of Bax in both groups was high the first day after PH, and decreased progressively until the end of the observation period; however, the expression of Bax in the NCPB-treated group was lower than that of control group at 3 and 7 days after PH (p<0.01) (**Fig. 7** **&** **Fig. 6** **A, C**). The expression level of B cell lymphoma/leukemia -2 (Bcl-2) in the control group was decreased until the 3 days after PH, and increased from then on. In the NCPB-treated group, Bcl-2 expression increased progressively after PH, and was higher than that of the control group at 3 and 7 days after PH (p<0.01) (**Fig. 8** **&** **Fig. 6** **A, D**).

### Hepatic Expression of NF-κB p65 and c-Jun

**Figure 9:**
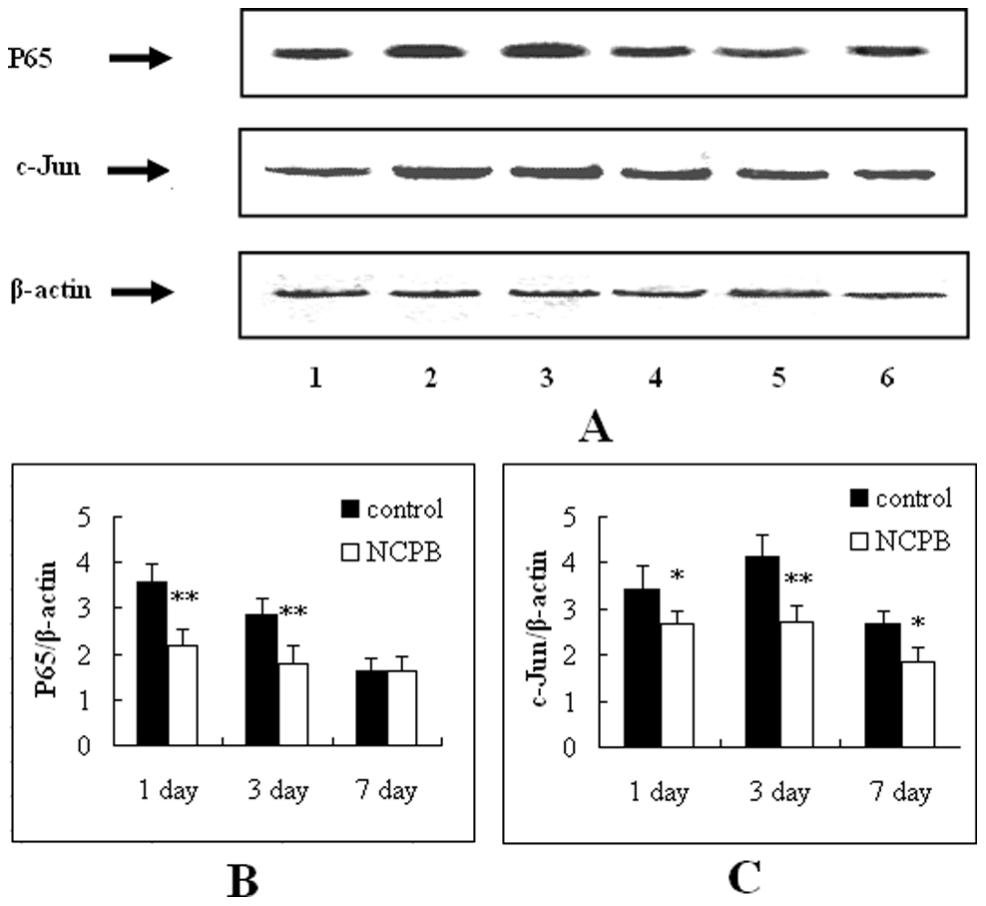
Protein expression level of NFκB p65 and c-Jun proteins in the liver after PH. (A) Lanes 1–3 represent the protein expression level in the control group at 7, 3 and 1 days after PH, respectively. Lanes 4–6 represent the protein expression level in NCPB group at 1, 3 and 7 days after PH, respectively. The expression of NF-κB p65 and c-Jun were detected by Western blot analysis and normalized to response to β-actin. (B–C) represent the statistical charts of NF-κB p65 and c-Jun proteins expressions in the liver after PH, respectively, and asterisks indicate significant differences from control group. *p<0.05; **p<0.01.

The protein expression level of NF-κB p65 and c-Jun was determined by Western blotting (**Fig. 9A**). The protein expression level of NF-κB p65 in the control group decreased progressively after PH, whereas its expression in the NCPB group decreased until 3 days after PH, and then increased. Additionally, the expression level of NF-κB p65 in the NCPB group was significantly lower than that of the control group at 1 and 3 days after PH (p<0.01; **Fig. 9B**). The protein expression level of c-Jun was also significantly lower in the NCPB group compared to the control group at each time point after PH (p<0.01; **Fig. 9C**). The relative protein level was normalized to the Western blot intensity of β-actin.

## Discussion

The liver is an important organ for the synthesis and secretion of plasma proteins, including albumin, fibrinogen, blood coagulation factors, and CRP. It is well established that the liver can regenerate itself following liver injury, including therapeutic PH. The current study demonstrates that NCPB has a potent protective effect against mortality in rats following PH; then, we discuss the results of experiments designed to understand the mechanisms underlying this effect. To ensure the blood supply of the heart, brain and other vital organs, the redistribution of the body’s blood and vasoconstriction of visceral vessels was induced, causing ischemia and hypoxia resulting in damage to the residual liver tissues. In addition, vasospasm of perfused vessels in the residual liver tissue was ineluctably caused by the liver surgery, causing decreased hemoperfusion of the residual liver tissues. The obvious stress reactions induced by pain and bleeding after PH, and the hypothalamic-pituitary-adrenal axis and sympathetico-adrenomedullary system over-excited, caused an increase in catecholamine secretion by adrenal medulla.

Our results show that the volume of blood flow in the liver of the control group was lowest at 1 day after PH, and the color of the hepatic tissues were pale, which is consistent with the effects of PH described above. Additionally, our results revealed that the blood supply of the residual liver tissue can be improved by treating with NCPB, which is a possible mechanism for the protective effects of NCPB treatment on regeneration of the residual liver after PH. Reconstruction of the sinusoidal vascular network is an important process during liver regeneration, as it not only supplies blood to the liver, but also promotes reconstruction of the liver structure. VEGF is a strong and specific vascular endothelial cell growth factor, and has been shown to be important and indispensable for liver regeneration [10], [11]. It has been reported that proliferating liver cells after PH can secrete VEGF which is needed to stimulate growth of the sinusoidal endothelial cells, and regulate the proliferation of hepatic sinusoidal endothelial cells by up-regulation the VEGF receptor. VEGF can not only stimulate and regulate the proliferation and migration of endothelial cells, but it can also significantly increase vascular permeability, and induce the liver to secrete and activate collagenases and blood plasminogen; therefore, the formation of capillary plexus and reconstruction of hepatic sinusoid were induced; and finally, the reconstruction of liver was completed [12], [13]. VEGF is not only a growth factor required for stimulating endothelial cell proliferation, but also a protection factor for endothelial cells under apoptosis [14]. Our results show that the expression of VEGF in the residual liver tissue was very low at 1 day after PH, and increased progressively with the proliferation of liver cells; however, the VEGF expression in the residual liver tissue of NCPB group can become significantly elevated rapidly, which indicated that NCPB treatment can rapidly promote the proliferation & migration of vascular endothelial cells and the formation of neonatal capillary plexus, and improve the blood supply of the residual liver tissues by increasing the expressions of VEGF, accordingly strengthening the liver regeneration. What’s more, our results demonstrated that the levels of circulating AST and ALT can be significantly decreased by treating with NCPB; however, the TB concentration can only be decreased 3 days after PH. The levels of AST and ALT can reflect the extent of hepatocyte injury [15], thus our results show that NCPB treatment can alleviate hepatocytes damage after PH.

CRP is an acute phase reactive protein, which is a principal member of the first defense line of the host natural defenses; therefore, the stress reaction extent can be evaluated by measuring CRP [16]. Our results show that the circulating CRP level of PH rats can be decreased by NCPB treatment, which indicates that the stress response induced by PH can be alleviated by treating with NCPB. Inflammatory cytokines, including TNF-α, IL-1β and IL-6, can be released by activation of mononuclear macrophages after severe trauma, infection and shock. Inflammatory cytokines, including TNF-α, IL-1β, IL-6 and IL-10 were also important inflammatory mediators for the development of SIRS and immune function disorders [17], [18]. Therefore, the severity of SIRS can be evaluated directly by changes in these inflammatory cytokines. Our results revealed that NCPB treatment can decrease the secretion of the inflammatory cytokines such as TNF-α, IL-1β and IL-6, decreasing the severity of SIRS.

It has been established that the MAPK and JAK/STAT pathways play important roles in the expression of inflammatory cytokines [19]. Furthermore, the phosphorylation of MAPK and JAK/STAT can further activate the NF-κB and AP-1 signaling pathways, initiating the transcription of cytokines and mediators of inflammation, and finally, leading to organ dysfunction. In view of the importance of the NF-κB and AP-1 signaling pathways in this inflammatory reaction, the effects of NCPB treatment on this pathway were studied, thus helping to clarify the molecular mechanisms of NCPB-mediated inhibition of SIRS after PH. NF-κB is an important inflammatory transcription factor, regulating the expression of many genes; its expression is known to correlate with proliferation, differentiation, and apoptosis. Additionally, the p65/p50 heterodimers were the common and active style in NF-κB. Our Western blotting analysis shows that the overexpression of NF-κB p65 can be significantly inhibited by treating with NCPB, which indicated that NCPB’s effect on the NF-κB pathway may underlie the protective effects of NCPB on the development of SIRS after PH. AP-1 consists of a Fos/Jun heterodimer or a Jun/Jun heterodimer, with the most common form of AP-1 being c-Jun/c-Fos. Signaling through AP-1 mediates cell proliferation, differentiation and expression of inflammatory cytokines [17]. The inflammatory proteins regulated by AP-1 include TNF-α, IL-1, IL-6, IL-8, MIP-1α, MCP-1, ICAM-1, VCAM-1 and iNOS. It is also known that increased AP-1 activity can be induced by increased expression and activity of c-Jun [20], [21]. The expression of c-Jun in NCPB-treated rats was significantly inhibited after PH in our study, which indicates another possible molecular mechanism through which NCPB protects against the development of SIRS after PH.

The excessive apoptosis of residual liver cells after PH can be induced by secondary injury factors, leading to functional lesions. The apoptosis-related genes Bcl-2 and Bax are two important members of the Bcl-2 family; they are widely distributed in body and can regulate apoptosis. In our study, the expression of Bax in NCPB-treated rats was significantly down regulated at 3 and 7 days after PH, whereas the expression of Bcl-2 was increased. These results indicate that the pro-apoptotic mechanism of liver cells can be started early after PH, and diminish progressively with liver tissue regeneration, whereas anti-apoptotic mechanisms are progressively increased. Our results show that this process can be accelerated by treatment with NCPB, and the expression of Bcl-2 can be up-regulated in the early phase to inhibit apoptosis. We believe this is an important reason for the improvement of liver functions following PH in rats treated with NCPB.

In conclusion, the present study demonstrates that NCPB treatment is associated with favorable outcomes and improved liver regeneration after PH. Based on these results, we suggest that NCPB can be utilized as an effective therapeutic method to improve outcomes following PH. However, more investigations are necessary to fully elucidate the mechanism of action of NCPB.

## Materials and Methods

### Ethics Statement

All animal treatments were strictly in accordance with international ethical guidelines and the National Institutes of Health Guide concerning the Care and Use of Laboratory Animals, and the experiments were carried out with the approval of the Animal Experimentation Ethics Committee of the General Hospital of Chengdu Military Command Area.

### Animals

The animals were provided by the medical laboratory animal center of the Third Military Medical University and followed the Principles of Laboratory Animal Care. Male Sprague-Dawley rats, weighing 200±20 g, were used in our study. Rats were kept on a 12-h light/dark cycle with free access to standard laboratory chow and water. Humidity was maintained at 50% and the temperature at 25°C. Each animal was used only once in the experiment.

### Chemicals and Reagents

Tissue total protein extraction reagent was purchased from Pierce (USA); Anti-NF-κB p65 rabbit polyclonal IgG, Anti-c-Jun rabbit polyclonal IgG, Anti-BAX rabbit polyclonal IgG, Anti-Bcl-2 Rabbit polyclonal IgG, and Goat Anti-VEGF monoclonal IgG were purchased from Santa Cruz Biotechnology, Inc. (USA). The Rabbit anti-goat IgG/HRP and Goat anti-rabbit IgG/HRP were obtained from Zhongshan Biotech Co. Ltd. (Guangdong, China). SDS-PAGE Molecular high weight markers for proteins were purchased from the Shanghai Lizhudongfeng Biotech Ltd. (Shanghai, China). The prestained protein molecular weight marker was purchased from Xian Runde Biotech Ltd. (Xian, China). Coomassie brilliant blue (G-250 and R-250), phenylmethylsulfonyl fluoride (PMSF), AP, and β-mercaptoethanol were purchased from Sigma-Aldrich Co. (USA). Dithiothreitol (DDT) was purchased from Gibco Co. (USA). The rat TNF-α ELISA kit, rat IL-1β ELISA kit, rat IL-6 ELISA kit, and rat C-Reactive Protein (CRP) ELISA kit were purchase from Cusabio Biotech Co. Ltd. (USA). All other chemicals used in this study were of analytical reagent grade.

### Partial Hepatectomy

Our rat PH model was prepared using the method described previously by Higgins and Anderson [22], with minor modifications. In brief, approximately 70% of the liver (left and middle hepatic lobe) was surgically removed from the anesthetized rat.

### Neurolytic Celiac Plexus Block

The method to achieve percutaneous NCPB in rats was performed as described previously [23], with minor modifications. In brief, 0.5% xylocaine was injected once the needle tip reached the anatomic site of the celiac plexus once per day, over a total of 7 days. For the control group, rats were underwent the same surgical procedure, but physiological saline was injected instead of 0.5% xylocaine.

### Experimental Design

A total of 70 rats underwent PH as described above. Thirty rats were equally divided into the following two groups (n = 15): (1) Control group, and (2) NCPB-treated group. For the NCPB-treated group, percutaneous NCPB was performed with 0.5% xylocaine after PH. Five rats were used to collect blood and liver tissues at each time point (1, 3, and 7 days), [under pentobarbital sodium (60 mg/kg)] after percutaneous NCPB. The serum samples were separated by natural deposition, and were stored at −70°C until further analysis. The weights of the total liver after PH were determined, and then the liver tissues were fixed with neutral formalin. For survival analysis, another 40 animals with PH were equally divided into 2 groups (n = 20), including one Control group and one NCPB-treated group. These animals were observed 1, 3 and 7 days after surgery, without liver or blood sampling (a humane endpoints evaluation was used in our experiment, and the body weight loss of 20% was used as the indicator).

### Determination of Hepatic Blood Flow

The hepatic blood flow of PH rats was determined by the radioactive microsphere method as described previously [24], [25]. Red blood cells from the toad, marked with 99mTc, were used as radioactive microspheres.

### Determination of Liver Function of Rats after PH

The automatic biochemistry analyzer (Siemens ADVIL 1650, Germany) was used to determine the concentration of alanine transaminase (ALT), aspartate transaminase (AST) and total bilirubin (TB) in serum.

### Determination of Serum CRP, TNF-α, IL-1β and IL-6 Concentration

The serum CRP, TNF-α, IL-1β and IL-6 concentrations were determined by ELISA and quantified using a microplate reader (Bio-Tek ELX800) at 450 nm with commercial kits.

### Histopathological Examinations

Tissue sections were dissected and fixed in neutral formalin. After fixation the samples were routinely processed, embedded in paraffin, sectioned at a 5 µm thickness, de-paraffinized, rehydrated and stained with hematoxylin and eosin [26]. The histopathological changes were evaluated in liver sections by light microscope (Olympus, Japan).

### Immunohistochemistry

The immunohistochemistry assay was performed followed the method described on the commercial kits to examine the expressions of Bcl-2, Bax and VEGF in the liver tissues.

### Western Blotting

Total liver protein was extracted, and equal amounts of protein (50 µg) were separated by SDS-PAGE, blotted on polyvinylidene difluoride membranes, and probed with anti-Bcl-2, anti-Bax, anti-VEGF, anti-NF-κB p65 rabbit polyclonal IgG and anti-c-Jun rabbit polyclonal IgG primary antibodies, following by incubation with a goat anti-rabbit/HRP secondary antibody, and detected by chemiluminescence. To measure protein loading, antibodies directed against β-actin were used.

### Statistical Analysis

All of the experiments were conducted at least in triplicate, and the data are presented as 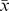
±*s*. The chi-squared of exact test was used to analyze the significance of rat mortality differences among groups. Statistical analyses were performed using the two-tailed Student’s t test with a significance level of p<0.05.

## References

[1] Garcea G, Ong SL, Maddern GJ (2009) Predicting liver failure following major hepatectomy. Digest Liver Dis 41: 798–806. 10.1016/j.dld.2009.01.015 19303376

[2] Fausto N (2000) liver regeneration. J Hepatol 32: 19–31. 10.1016/s0168-8278(00)80412-2 10728791

[3] Baue AE, Durhamm R, Faist E (1998) Systemic inflammatory response syndrome (SIRS), multiple organ dysfunction syndromes (MODS), multiple organ failure (MOF): are we winning the battle. Shock 10: 79–89. 9721973 10.1097/00024382-199808000-00001

[4] Marcu AC, Kielar ND, Paccione KE, Barbee RW, Csrter H, et al (2006) Androstenetriol improves survival in a rodent model of traumatic shock. Resuscitation 71: 379–386. 16982126 10.1016/j.resuscitation.2006.03.020

[5] Erdek M, Halpert DE, González Fernández M, Cohen SP (2010) Assessment of celiac plexus block and neurolysis outcomes and technique in the management of refractory visceral cancer pain. Pain Med 11: 92–100. 20002595 10.1111/j.1526-4637.2009.00756.x

[6] Wong GY, Schroeder DR, Carns PE, Wilson JL, Martin DP, et al (2004) Effect of neurolytic celiac plexus block on pain relief, quality of life, and survival in patients with unresectable pancreatic cancer: A randomized controlled trial. JAMA 291: 1092–1099. 14996778 10.1001/jama.291.9.1092

[7] Vorenkamp EK, Dahle AN (2011) Diagnostic celiac plexus block and outcome with neurolysis. Tech Region Anesth Pain Manage 15: 28–32.

[8] Steinman L (2004) Elaborate interactions between the immune and nervous systems. Nat Immunol 5: 575–581. 15164017 10.1038/ni1078

[9] Tracey KJ (2002) The inflammatory reflex. Nature 420: 853–859. 12490958 10.1038/nature01321

[10] Bockhorn M, Goralski M, Prokofiev D, Dennis P, Grunewal P, et al (2007) VEGF is important for early liver regeneration after partial hepatectomy. J Surg Res 13: 291–299. 10.1016/j.jss.2006.07.027 17275844

[11] Michalopoulos GK, Defrances MC (1997) Liver regeneration. Science 276: 60–66. 9082986 10.1126/science.276.5309.60

[12] Shimizu H, Mitsuhashi N, Ohtsuka M, Ito H, Kimura F, et al (2005) Vascular endothelial growth factor and angiopoietins regulate sinusoidal regeneration and remodeling after partial hepatectomy in rats. World J Gastroenterol 11: 7254–7260. 16437624 10.3748/wjg.v11.i46.7254 PMC4725143

[13] Greene AK, Wiener S, Puder M, Yoshida A, Shi B, et al (2003) Endothelial-directed hepatic regeneration after partial hepatectomy. Ann Surg 237: 530–535. 12677150 10.1097/01.SLA.0000059986.96051.EA PMC1514466

[14] Alavi A, Hood JD, Frausto R, Stupack DG, Cheresh DA (2003) Role of Rafin vascular protection from distinct apoptotic stimuli. Science 301: 94–96. 12843393 10.1126/science.1082015

[15] Omari A, Bani-Hani KE (2007) Effect of carbon dioxide pneumoperitoneum on liver function following laparoscopic cholecystectomy. J Laparoendosc Adv Surg Tech A 17: 419–424. 17705719 10.1089/lap.2006.0160

[16] Sjowall C, Wettero J (2007) Pathogenic implications for autonatibodies against C-reactive protein and other acute phase peoteins. Clin Chim Acta 378: 13–23. 17239838 10.1016/j.cca.2006.12.002

[17] Li J, Liu YH, Ou S, Dai XM, Wang JP, et al (2012) Steroid receptor coactivator-3 differentially regulates the inflammatory response in peritoneal macrophages. Mol Med Rep 5: 1099–1105. 22245955 10.3892/mmr.2012.750 PMC3493053

[18] Li J, Niu J, Ou S, Ye ZY, Liu DQ, et al (2012) Effects of SCR-3 on the immunosuppression accompanied with the systemic inflammatory response syndrome. Mol Cell Biochem 364: 29–37. 22198336 10.1007/s11010-011-1201-y

[19] Carter AB, Monick MM, Hunninghake GW (1999) Both Erk and p38 kinases are necessary for cytokine gene transcription. Am J Respir Cell Mol Biol 20: 751–758. 10101008 10.1165/ajrcmb.20.4.3420

[20] Karin M, Li ZG, Zandi E (1997) AP-1 function and regulation. Curr Opin Cell Biol 9: 240–246. 9069263 10.1016/s0955-0674(97)80068-3

[21] Li J, Liu YH, Ye ZY, Liu HN, Ou S, et al (2011) Two clinically revevant pressures of carbon dioxide pneumoperitoneum cause hepatic injury in a rabbit model. World J Gastroentero 17: 3652–3658. 10.3748/wjg.v17.i31.3652 PMC3180024 21987614

[22] Higgins GM, Anderson RM (1931) Experimental pathology of the liver. I. Restoration of the liver of the white rat following partial surgical removal. Arch Pathol 12: 186–202.

[23] Jiang CL, Zhang LH, Wu YF, Qu XL, Cui YR, et al (2008) Establishment of model treated for neurolytic celiac plexus block percutaneously with anhydrous-alcohol in rat. Chin J Pain Med 14: 233–235.

[24] Bauer B, Walter B, Wurker E, Kluge H, Zwiener U (1996) Colured microsphere technique as a new method for quantitative-multiple estimation of regional hepatic and portal blood flow. Exp Toxic Pathol 48: 415–420. 10.1016/S0940-2993(96)80051-0 8765686

[25] Xie ZZ, Liu FY, Huang QY, Luo G, Zhang GB (1997) Effect of hypoxia on maximal myocardial blood flow in right ventrlcle. Chin J Appl Physiol 13: 302–305. 10322953

[26] Wang Y, Han T, Xue LM, Han P, Zhang QY, et al (2011) Hepatotoxicity of kaurene glycosides from xanthium strumarium L. fruits in mice. Pharmazie 66: 445–449. 21699085

